# Challenges of health services related to the population displaced by violence in Mexico

**DOI:** 10.11606/S1518-8787.2018052017094

**Published:** 2018-07-13

**Authors:** María Beatriz Duarte-Gómez, Silvia Magali Cuadra-Hernández, Myriam Ruiz-Rodríguez, Armando Arredondo, Jesús David Cortés-Gil

**Affiliations:** I Instituto Nacional de Salud Pública. Centro de Investigación en Sistemas de Salud. Cuernavaca, Morelos, México; II Universidad Industrial de Santander. Escuela de Medicina. Departamento de Salud Pública. Bucaramanga, Santander, Colombia; III Universidade Federal de Pelotas. Programa de Pós-Graduação em Epidemiologia. Pelotas, RS, Brasil

**Keywords:** Transients and Migrants, Minority Groups, Violence, prevention & control, Exposure to Violence, Social Vulnerability, Health Vulnerability, Social Inequity, policies, Review, Migrantes, Grupos Minoritarios, Violencia, prevención & control, Exposición a la Violencia, Vulnerabilidad Social, Vulnerabilidad en Salud, Inequidad Social, políticas, Revisión

## Abstract

**OBJECTIVE:**

To analyze the impacts of the care to the population displaced by violence on the health system and the challenges that this entails.

**METHODS:**

This is a narrative review of the national and international literature in PubMed, SciELO, WHO/PAHO, and Bireme. Inclusion criteria were date of publication (from 2000), relation with the subject, and language (Spanish or English). We found 292 documents, of which 91 met the inclusion criteria.

**RESULTS:**

The main challenges are the intersectoral, participatory, and integral approach (with emphasis on mental health and sexual and reproductive health), ensured accessibility to health services, the need for a reliable registration and information system of the population displaced by violence and its characteristics, and the addressing of the biopsychosocial problems of the different groups, especially women, persons with disabilities or infectious diseases, adolescents, children, ethnic minorities, older adults and the lesbian, gay, bisexual, transsexual, and intersexual population.

**CONCLUSIONS:**

The lack of political will to accept and see the internal displacement by violence and its importance as a humanitarian and public health problem is an obstacle to the adequate and timely care of the population displaced by violence in Mexico.

## RESUMEN

**OBJETIVO:**

Analizar las implicaciones de la atención de la población desplazada por violencia para el sistema de salud y los retos que le implica.

**MÉTODOS:**

Revisión narrativa de literatura nacional e internacional en PubMed, SciELO, WHO/OPS, y Bireme. Los criterios de inclusión fueron, la fecha de publicación (a partir de 2000), la relación con el tema, y el idioma (español o inglés). Se encontraron 292 documentos, de los cuales 91 cumplían con los criterios de inclusión.

**RESULTADOS:**

Los principales retos se referirán: al enfoque integral (con énfasis en la salud mental y la salud sexual y reproductiva), intersectorial, y participativo; a la garantía de la accesibilidad a los servicios de salud; a la necesidad de un sistema de información y registro confiable de la población desplazada por violencia y sus características; y al abordaje de las problemáticas biopsicosociales de los diferentes grupos, especialmente mujeres, personas con discapacidad o con enfermedades infecciosas, de adolescentes, niños y niñas, minorías étnicas, población adulta mayor y la población lésbica, gay, transexual, bisexual y intersexual.

**CONCLUSIONES:**

La ausencia de voluntad política para la aceptación y visibilización del desplazamiento interno por violencia y de su importancia como problema humanitario y de salud pública, constituye un obstáculo para atender a la población desplazada por violencia de forma adecuada y oportuna en México.

## INTRODUCTION

A displaced population is defined as “individuals or groups of people who have been forced to flee their homes to escape armed conflict, generalized violence, and human rights abuses”^1^. It includes refugees – persons who crossed an international border – and internally displaced persons. This displacement becomes a public health problem^2^ ^,^ ^3^ and a challenge for the provision of health services (HS) in both the sending and receiving areas, many of which do not have the resources to respond to a sudden and massive influx of persons^4–6^.

A displaced population faces a forced and abrupt transition that involves individual and family changes, both in their roles or relationships and in their material living conditions. This usually means shortages of resources, family conflicts, health risk behaviors, sexual abuse, physical and mental health problems, and human rights violations^7^. Displaced populations mainly have low and very low socioeconomic status (SES), which does not mean that persons with high SES are not displaced or do not suffer when they do so, but they can rely on greater cultural and economic capitals to face it^8^. Quality of life tends to worsen with displacement, especially in the transition phase - between choosing a temporary settlement until they can be permanently placed^9^. At this stage, there is no more emergency aid, and thus the economic situation often deteriorates^10^ ^,^ ^11^. The arrival of a new population affects the receiving area in relation to the labor market, security, and demand for public services. This sometimes leads to rejection and discrimination and the need to mobilize new resources at the state and city level^3^ ^,^ ^12^ ^,^ ^13^.

Official statistics on forced displacement are scarce and incomplete in Mexico. The internally population displaced by violence has increased in the country, especially in states such as Chihuahua, Michoacán, Sinaloa, Durango, and Guerrero^3^ ^,^ ^14^. In Mexico, 19,747,511 persons migrated internally in 2010, according to the 2015 data of the National Institute of Statistics and Geography (INEGI)^15^. The most frequent reasons were poverty, violence, and natural disasters^16^. According to the 2014 National Demographic Dynamics Survey, six out of 100 migrants moved to another state motivated by public insecurity or violence^17^. According to the latest National Survey of Victimization and Perception on Public Security (ENVIPE) in 2014, 1.6 million persons migrated internally because of violence^18^. The study of Chávez and Warnner^19^, in 2012, estimates that Mexico has half a million internal migrants, with differences according to education level (lower education level plus group and rural migration) and age (younger individuals migrate more), with little difference by sex. However, they do not differentiate the cause of migration; they only mention Chihuahua and Sonora as sending areas, with little immigration and problems of organized crime.

Mexico had high internal migration for economic reasons, mainly from the south to the north of the country. It is also a transit point for Central and South American migrants to the United States^20^. However, it has not officially recognized the existence of internal migration by violence as a priority problem. Consequently, it is little visible, there is no adequate information, and the responses to this phenomenon were fragmented and insufficient, as well as the policies and programs for this population^21^. The Government must ensure the right to health, in a holistic conception, that goes beyond the medical care of the disease. This reluctance to acknowledge the existence of displaced populations hinders the care provided to them^22^.

Given the exposed problem, the objective of the study was to analyze the impacts of the care to populations displaced by violence to the health system and the challenges that this entails.

## METHODS

This first approach was based on the identification and analysis of the national and international literature, which contributes with information for the formulation of public policies that allow the preparation, allocation of resources, design, and implementation of national programs that respond to the needs of this vulnerable population.

We carried out a narrative review of the literature on forced migration associated with violence and the response of health systems in documents published from 1995 to 2016. We included studies with different methodologies, from any country. The searches in PubMed, SciELO, WHO/PAHO, and Bireme were performed under the following terms in English and Spanish:

- Health services and/or Health system and internal displacement by violence;
- Displaced population and health services;
- Forced displacement and health in Mexico.

Inclusion criteria were date of publication, relation with the subject, and language (Spanish or English). Each document was classified by document type, geographical context, and category in relation to the subject (Table).

**Table:** Classification of information found in the 1995–2016 literature review on displaced population and health services.

Criterion	Type	Articles (n)
According to type of source	•Scientific articles •Technical reports from agencies and NGO and theses •International Agencies UNHCR, IOM, NGO •National Agencies •Plans, programs, legislation •Manuals, guides	47 30 9 7
According to geographical context	•Mexico •Latin America •Other	7 51 35
According to components of Health Systems	•Human resources •Infrastructure/Equipment •Organization/Operation •Financing/Costs •Other	17 9 23 16 41
According to relation with the subject	•Directly related: those that explicitly talk about health services for a displaced population •Indirectly related: those whose main subject is the characteristics of this population and health needs, which is indirectly related to the services that must be provided to meet them •Unrelated: those whose main subject is not related either to health services or to the needs of the displaced population	42 51 201

NGO: non-governmental organization; UNHCR: United Nations High Commissioner for Refugees; IOM: International Organization for Migration

We found 292 documents, of which 91 met the inclusion criteria. Most (n = 75) were from the Latin America, almost all from Colombia. Of the 11 documents from Mexico, two were directly related to health services^23^ ^,^ ^24^ (Figure).

**Figure:**
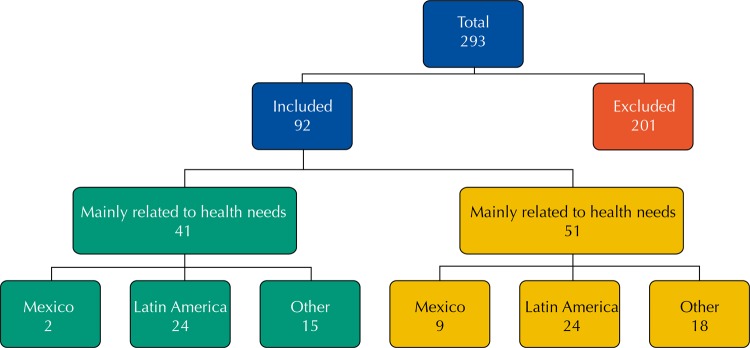
Number and type of documents selected in the 1995–2016 literature review on displaced population and health services.

## RESULTS

### Forced Displacement and Health Services

We found scarce specific literature on the effect of forced displacement on HS. The articles related to the subject have identified its potential negative impact on human resources, as well as the increase in the needs of drugs, inputs, and infrastructure in health services^2^.

A study on health and forced displacement in Sri Lanka, although focusing on post-conflict, has found three documents on HS. Most focused on the mental health problems of the displaced population^25^.

The article of the International Center for Migration and Health^26^ has highlighted the areas that should be the focus of health policies for this population: communication, infection control, maternal and child health, occupational health, violence, creation of health indicators, and staff preparation for the intercultural capabilities needed for the care of ethnic minority groups. Principle 19 of the “Guiding Principles on Internal Displacement” of the United Nations prioritizes the care of the displaced population with disabilities, infectious diseases, and women, especially in the field of sexual and reproductive health (SRH)^1^. The World Health Organization (WHO)^2^ mentions that the public health should avoid inequities between the displaced population and the host population regarding the health condition and access to HS. To this end, it proposes that health rights should be ensured without discrimination and obstacles should be removed for preventive and curative interventions that reduce excess morbidity and mortality and minimize the consequences both on community health and cohesion.

Some studies have focused on barriers to access to services. The most mentioned ones were the geographic, economic, cultural, and bureaucratic barriers^5,12,27–30^. Some strategies used in Colombia to ensure access were the priority affiliation to social security or the portability of this affiliation, the flexibility to adapt to obstacles such as the frequent absence of identification document, and the care with mobile health brigades and managers^29,31–33^.

Others have mentioned the lack of staff training to handle social and health emergencies, aggravated by the high turnover from the work under stress conditions, lack of resources, and insecurity^34^. They also have highlighted local shortcomings in developing policies to support the displaced populations, whether for lack of interest, ability, incentives, or funding^12^ ^,^ ^3^ ^5^. Most of the experiences came from Colombia, where a care model was designed for displaced populations, based on mobile teams focused on Primary Health Care (PHC) and specific manuals^4^ ^,^ ^36^.

There is a consensus about the characteristics that the HS should have for displaced populations, such as cultural acceptability^2,5,26,37–39^, integrality^24^ ^,^ ^40^, appropriate information system^4,41–43^, being it intersectoral^4,12,29,44–47^, participatory^5^ ^,^ ^29^ ^,^ ^33^, and accessible^24,27–31,37,43,48–5^ ^1^, and with security measures that ensure the lives of both the population and health personnel^1^ ^,^ ^12^ ^,^ ^34^ ^,^ ^44^ ^,^ ^52^ (Box 1).

**Box 1:** Characteristics of the health services for population displaced by violence, found in the 1995–2016 literature review on displaced population and health services.

Characteristic	Definition	Authors
Cultural acceptability	It implies adaptations in terms of communication and organization when the displaced population is indigenous or comes from another region culturally distinct from the receiving area	Juliao-Vargas^37^, Lattay Goodman^38^, OMS^93^, Thomas y Thomas^5^, Kinzie^39^, Siem^26^
Integrality	To know how to identify and meet the physical and psychosocial health needs, which implies trained human resources	Reynolds^24^, UNICEF^40^, Ruiz Rodriguez et al.^49^, Galambos et al.^95^, Vilar Peyrí y Eineschutz Hartman^69^, Diez-Ruiz y Agudelo Suarez^70^, Eisenman et al.^71^, OMS^72^, Juliao-Vargas^37^, Rodríguez et al.^84^, OMS^54^, OMS^85^, OMS^94^, Betancourt y Castro^86^, Cohen^73^, Cámara de Diputados^91^
With appropriate information system	To allow the differentiated care of the population (according to sex, age, ethnicity, health status) and the organized allocation of resources	Alcaraz López et al.^41^, IASC^42^, Hernández y Gutiérrez^43^
With intersectorial coordination	To meet basic needs (sanitation, housing, food, education, security)	Balladelli^29^, Bernal Pulido^12^, OPS^44^, Schuster y Schwendke^45^, Rodríguez Neyra^4^, Silva^46^, Fox et al.^47^
Participatory	To promote and enable the active involvement of the population in the decision-making of programs and strategies	Balladelli^29^, OPS^33^, Ministerio de Protección Social^48^, Thomas y Thomas^5^
Accessible	With geographical, functional, economic, and cultural access	Ruiz^28^, Ruiz^49^, Ministerio de Salud^29^, Hernández^43^, Juliao^37^, Cáceres^50^, Vidal^31^, Mogollón^30^, Ballesteros-P et al.^27^, Reynolds^24^, Moreno et al.^51^, OMS^94^, OPS^32^, OPS^33^
With security measures	Both for displaced population and health personnel	Bernal Pulido^12^, Organización de las Naciones Unidas^1^, OPS^44^, Kalipeni y Oppong^34^, Paho^52^

OMS: World Health Organization; IASC: Inter-Agency Standing Committee; PAHO: Pan American Health Organization; OPS: Pan American Health Organization

Other factors have also been identified on the challenges for the HS:

- The amount of displaced populations: individual or family (drop by drop) or massive (more than 10 families or 50 persons)^21^ ^,^ ^5^ ^3^.
- The existing resources in the receiving area and previous planning. According to the WHO, the political and security conditions of a country allow predicting possible displacements, planning activities and resources, and creating protocols for when the situation occurs^5^ ^,^ ^54^.
- The displacement stage: initial (addressed as a health emergency) or in the final settlement stage^4^ ^,^ ^21^ ^,^ ^51^ ^,^ ^55^.
- Population type: minority ethnic groups or population similar to the receiving area^37^. Most displaced populations in the Latin America are ethnic minorities (indigenous and Afro-descendants)^14,56–58^.
- The age groups of the displaced population and their health status^1,5,27,28,47,59–67^, with emphasis on childhood, adolescence, adult women, minority ethnic groups, the lesbian, gay, bisexual, transsexual, and intersexual population (LGBTI) population, and persons with disabilities or chronic or infectious disease requiring long-term treatment, such as TB and AIDS (Box 2).
  **Box 2:** Priorities of care according to age group in displaced population, found in the 1995–2016 literature review on displaced population and health services.

Age group	Priorities	Authors
Childhood	Because of the vulnerability of this group to food shortages, health conditions, changes in residence, and post-traumatic stress disorder (PTSD) and its consequences, it is recommended to emphasize on: continuity of maternal breastfeeding, vaccination, nutrition, detection and addressing of mental health problems with family and school interventions, and friendly health services	James et al.^59^, Kataoka et al.^60^, Patten^61^, Ruiz Rodriguez et al.^28^, Thomas and Thomas^5^, Fox et al.^47^, Gómez Builes et al.^56^, Ballesteros-P et al.^27^, Baquero Latorre et al.^62^, Cámara de Diputados^91^
Adolescence	Prevention of sexual or economic exploitation or violence, STI/HIV, pregnancy, addictions, and PTSD	Thomas and Thomas^5^, Salama and Dondero^63^, Zea et al.^64^, Yearwood et al.^65^, Mogollón Pérez e Vásquez Navarrete^66^, Cámara de Diputados^91^
Adult women	Psychosocial care of pregnant women and mothers head of the family	Mogollón Pérez and Vásquez Navarrete^66^, Organización de las Naciones Unidas^1^, Ballesteros-P et al.^27^, Roberston et al.^68^

PTSD: post-traumatic stress syndrome; STI/HIV: sexually transmitted infections/HIV

Since most displaced populations have a low educational level^19^ ^,^ ^62^, they require information on health, rights, and services, to improve their use, especially on SRH (prevention and detection of cervical and breast cancer, sexually transmitted infections/HIV, and family planning)^55^.

### Health Needs of the Displaced Population

Given the lack of specific information about the challenges to HS, they can be deduced from the health information of the displaced population, which is abundant. Although the conditions of forced migration by violence in other continents are different because of their magnitude and socio-political context, there are common characteristics in displaced populations: most are poor, women, with low education level, with traumatic experiences and stress, placed in areas with poor health conditions, with few belongings, and often without identity documents^5^.

Most references have identified mental health problems^42,44,50,60,68–^ ^72^ from stress and the accompanying trauma of displacement, often preceded by murders, threats, violations, and losses. To this, we can add the stress in the receiving area, from unemployment, discrimination, and loss of networks. The most common problems were depression and post-traumatic stress syndrome (PTSD), whose magnitude depends on personal factors and the environment^5,57,71–73^.

The second most mentioned subject was the SRH needs of women, especially teenagers, because of the risk of sexual abuse and exploitation both at the receiving place and within the displaced group itself^74^ ^,^ ^75^. This involves efforts to identify and prevent unwanted pregnancy, STI/HIV, and unsafe abortion and to ensure the availability of contraceptives, including emergency contraceptives, and antiretrovirals^1^ ^,^ ^5^ ^,^ ^73^ ^,^ ^75^ ^,^ ^76^.

Other groups mentioned were older adults^53^ and persons with disabilities^1^ ^,^ ^53^. The LGBTI population was considered important because of the discrimination they may be subject to, even in the HS, and those with STI/HIV treatments to ensure continuity^1^ ^,^ ^77^. Hence the importance of having an epidemiological surveillance system^63^ that ensures timely diagnosis and continuity of treatment, as well as the diagnosis of available resources. We found some articles on specific subjects: increased canine rabies in displaced areas^78^, oral health^79^ ^,^ ^80^, and occupational health^26^ ^,^ ^81^.

### Components of the HS

The PAHO comments that a massive internal displacement produces an increased magnitude and distribution of the burden of the disease and, therefore, increases the volume and composition of the demand for services^36^. Most documents mentioned one or more of the HS components:

- Infrastructure and equipment: in addition to the resources for emergency humanitarian care, health care actions are contemplated as part of the integral care of the victims. They can be individual (prosthesis, physical and mental rehabilitation) or collective (provision of equipment and inputs, construction of health centers)^82^ ^,^ ^83^. Massive displacement can cause shortages of care resources in the absence of appropriate preparation for these situations^30^.
- Human resources (HR). The health staff needs to be trained on the care of displaced populations^5^ ^,^ ^54^, as well as state and city authorities. Adequate remuneration and emotional restraint are important in the initial stages of emergency, followed by security measures^26^ ^,^ ^36^ ^,^ ^82^. International agencies have designed manuals for emergency care and for mental health problems in this population^36^ ^,^ ^44^ ^,^ ^72^ ^,^ ^84^ ^-^ ^86^.
- Reinforcement of services: in particular SRH^5^ ^,^ ^75^ ^,^ ^76^ and mental health^42^ ^,^ ^55^. In Colombia, the Integral Care System for Population Displaced by Violence details the HS required. The services range from prevention and emergency care up to psychosocial relocation and rehabilitation^48^.
- The financing of health care for displaced populations was a scarce subject in the documents. There was a consensus that financial resources are scarce or late^30^ and national budget actions are needed so that the local population is not negatively affected^12^. Other texts mentioned funding sources^29^ ^,^ ^87^, payment mechanisms^29^ ^,^ ^86^ ^,^ ^88^, or the budget allocated for the reparation of victims^35^. However, no article has done a detailed study of health costs derived from displacement^89^. Given that displaced families are poor and should also abandon their sources of income, their ability to pay is minimal, even for transportation to the health unit^5^ ^3^. The WHO highlights the persistent scarcity of resources for mental health care, a priority service for displaced populations^85^.
- Other components: different subjects have been addressed, such as intersectoriality, participation, and empowerment of the displaced population^4^ ^,^ ^29^ ^,^ ^55^ ^,^ ^82^, the role of non-governmental organizations^14^ ^,^ ^2^ ^1^ ^,^ ^55^, and the role of the academy^28^ ^,^ ^35^ ^,^ ^41^ ^,^ ^62^ ^,^ ^80^ both in care and in research.

### The Situation in Mexico

Despite underreporting, 281,418 persons are known to be victims of internal displacement forced by violence, mainly in the Northern states and Guerrero, caused by organized crime^21^ or religious problems. In general, the subject of displaced populations has been little studied and recognized, with no specific policies or legal framework in the field of health services^23^ ^,^ ^53^. An exception is Chiapas, where a law was passed to protect the rights of this population^90^. There are state legislations in Guerrero and Sinaloa^14^. The Federal Project for the Care of Displaced Indigenous People does not include health aspects, but it recognizes both the existence of an indigenous population displaced by some type of violence and the lack of specific legislation that recognizes and characterizes it^58^. Other state responses include emergency response and intersectoral committees (Guerrero) and employment programs (Sinaloa, Durango). The General Victim Law of 2013^91^, which allowed the registration of victims, does not operate in all states; its Executive Committee does not have enough budget or service protocols or state laws for the Prevention and Care of Internal Displacement, which includes the right to medical services^14^ ^,^ ^21^ ^,^ ^90^. The initiative of a Federal Law for Internally Displaced People, introduced to the Chamber of Deputies in 1998, was dismissed.

The Arana document on the situation in Chiapas highlights the lack of information to identify population groups and needs for their care, and it reinforces the connection between forced displacement and health^23^:

“Forced displacement is one of the clearest examples of the interdependence between rights. Violation of civil rights of security and free residence from the use of physical and/or psychological force to expel a population unleashes an unpredictable series of negative consequences that hinder the exercise of the right to health and a long list of economic, social, and cultural rights.” (p.79)

## DISCUSSION

Forced displacement is an issue that violates human rights. It produces highly frail individuals and groups from a profound deterioration in the quality of life and health and the lack of care by the Government against the right to health^56^. We found no specific studies that have aimed to analyze the impact of forced displacement on HS, which is probably more related to the redistribution of the same resources. The mental health and rehabilitation aspects, traditionally and precariously cared for in the health system, are still pending.

While most documents refer to migration by violence from one country to another, rather than internal migration, the diagnosis of the needs of the migrant population generally allows the deduction of the impact and challenges for the HS. In Latin America, most of the literature refers to Colombia, where the magnitude of the problem is enormous and has been assumed as a national priority. This has resulted in a reliable system of registration, strategies, and policies specific to this population and a large number of research and interventions have been carried out by the academia, cities, NGO, and international organizations. For this reason, the Colombian experience is recommended as an example for Mexico^53^. In Peru, reparations for victims include care for disabilities, full recovery of community mental health, capacity building, and HS infrastructure and training^89^. In Mexico, as long as the government does not recognize internal displacement by violence as a public health, human right, and national security violation, actions to mitigate it will have little political and financial viability. States and cities and the displaced population itself must face the resources and health problems from the displacement.

Based on the results, the main challenges for the Mexican health system would be^23–25,45,49,50,^ ^62^ ^,^ ^69^ ^,^ ^73^ ^,^ ^75^ ^,^ ^83^ ^,^ ^92^:

1. Integral, intersectoral, and participatory approach, which requires multidisciplinary teams that are sensitized and trained^4^ and political will at all levels.
2. Ensuring of the geographical, cultural, economic, and functional accessibility to the HS, taking into account the psychosocial particularities of the displaced population, such as psychosocial trauma (fear, pain, uncertainty) and lack of resources, as well as the limitations of the benefit plan of the Popular Insurance.
3. Presence of a reliable information and registration system for the displaced population and its characteristics, which allows prioritizing activities and groups, with the participation of community leaders that facilitate the approach of the population and the flow of information.
4. Analysis and adaptation of successful experiences of other Latin American countries for the care and reparation of the population displaced by violence.
5. Full addressing of the problems of different groups, especially women, and within them, young women, who, in addition to the specific SRH needs, face the risk of sexual violence and domestic violence because of the gender. In addition, many become the head of a family, thus requiring great psychosocial support.
6. Given the high vulnerability of the displaced population to mental health problems, this problem must be studied and addressed in order to avoid negative individual, family, and social consequences.

To address these challenges adequately, the Mexican government must recognize the existence of displaced populations to plan resources and design policies^23^ ^,^ ^53^; it must activate a comprehensive humanitarian strategy to promote their rights and design a specific legal framework for the protection of internally displaced persons, including the protection of their health^35^ ^,^ ^93^.

Forced displacement accumulates vulnerabilities from losses: political and social rights, family and social networks, and adverse social and health conditions that are present in the receiving areas. These situations increase the burden of the disease and pressure the demand for health services that respond to health needs, particularly mental health needs. The WHO warns about the consequences of conflicts for public health and health systems which may have a decreased ability to respond adequately to new demands^94^. The accumulation of vulnerabilities of displaced populations is a challenge for health systems regarding the implementation of policies for more sensitive services. In particular, this aspect becomes relevant in care models that have an explicit homogeneous benefit plan that affects a series of psychosocial health problems, which are excluded from the care. For example, mental health problems have not been adequately addressed in Colombia because the mental health services included in the benefit plan are not enough to meet the perceived need^49^. Managed care models have also shown that they are not sensitive to the care of mental health problems^95^.

Forced displacement is a determinant of social and health inequities. It is closely related to structural factors, such as economic distribution, land, human rights, and intermediate factors such as socioeconomic status and health system. This aspect exposes another challenge for health systems regarding the inter- and trans-sectoral work that favors the social mobilization of players other than those in the health sector to enhance interventions in health services. Health systems should be centered on more comprehensive strategies, such as Primary Health Care and Health Promotion, and focused on Social Determinants, which have close relations.

Knowledge about the health problems and needs of the displaced population and its impact on the HS can be used to define programs, strategies, and policies that address this growing problem in Mexico. National and international experiences are an important reference for the country. Colombia has a broad trajectory in this area and has a National System of Integral Care for displaced populations and specific strategies of health care. Strategies include the training of health managers and mobile brigades, in addition to those designed and evaluated with the follow-up of the Pan American Health Organization^33^, with funding defined for each sector, a national registry of victims, and promotion of community participation in decision-making. This could be used by the Mexican Government^29^.

Despite the limitation of the scarcity of specific information on the subject, as shown in the Table, the analysis of the data found allows us to conclude that the Health System must respond to the needs of the different displaced groups and to the multiethnic and multicultural nature of Mexico with integral focus and rights, and with an anticipatory nature to avoid improvisation. The lack of political will to accept and see the internal displacement by violence and its importance as a humanitarian and public health problem is an obstacle to assist adequately and timely the population displaced by violence in Mexico. It is the responsibility of the three levels of the government to care for displaced populations and the redeployment of resources between sending and receiving areas and the awareness and training of health personnel for work in social and health emergency situations. Given the vulnerability characteristics of displaced populations, Mexico needs to develop a registry and a diagnosis of the physical and emotional health problems of this population to mitigate the suffering and the consequences in the short and long term. The component of social and community participation, which is present in primary health care and health promotion, is a fundamental element to ensure the right to health of the displaced population. Quantitative and qualitative research on the effect of internal displacement forced by violence on the HS in Mexico is needed to provide information for the planning of the strategies and resources needed for the health care of the growing displaced populations.
